# Use of Dietary Supplements among Spanish Pediatricians in Daily Practice: A Cross-Sectional Survey Study

**DOI:** 10.1155/2019/5819305

**Published:** 2019-07-24

**Authors:** Ignacio Güemes Heras, Alicia Santamaría-Orleans, José F. Colinas Herrero, Pilar Gómez Sorrigueta, Luis Ortiz González, Raquel de la Iglesia-Arnaez, Alejandro Canals Baeza

**Affiliations:** ^1^Department of Pediatrics, Hospital Católico Universitario Casa de Salud, Valencia, Spain; ^2^Department of Scientific Communication, Laboratorios Ordesa, Sant Boi de Llobregat, Barcelona, Spain; ^3^Service of Pediatrics, Centro de Salud García Lorca, Burgos, Spain; ^4^Service of Pediatrics, Centro de Salud El Crucero, León, Spain; ^5^Pediatrics Clinic Dr. L. Ortiz, Badajoz, Spain; ^6^Service of Pediatrics, Centro de Salud Alicante Santa Faz, Alicante, Spain

## Abstract

A cross-sectional survey study was designed to gather information on the use of dietary supplements by Spanish pediatricians. The study questionnaire was completed by 433 pediatricians (62% men, mean age 52.5 years) throughout the country. They also provided data on 10 of their patients (*n* = 4304) in which synbiotics, immune stimulants, and omega-3 polyunsaturated fatty acids (PUFAs) had been prescribed. Synbiotics were used by 92% of pediatricians, immune stimulants by 80.4%, and omega-3 PUFAs by 75.1%. Synbiotics were mainly used combined with antibiotics (92.6%) and for gastrointestinal disorders (91.2%), immune stimulants to enhance defenses and cold prevention (87.1%), and omega-3 PUFAs to improve symptoms of attention-deficit hyperactivity disorder (ADHD) (84.8%) and concentration (80.1%). Confidence and previous experience with the product (51.6%), composition and indications of the product (43.1%), and tolerability (39.9%) were main factors involved in decision-making. Children treated with omega-3 supplements were significantly older (mean age 7.6 (3.0) years) than those treated with synbiotics (3.9 (3.9) years) or immune stimulants (3.4 (2.8) years) (*P* < 0.001). Short duration of treatment (<1 month) was significantly more common in the synbiotics group (90.5%), whereas longer duration of treatment (>3 months) was more frequent in the omega-3 group (79.1%). In the immune stimulants group, 60.4% of patients were treated for a period between 1 and 3 months. Clinical improvement was rated by participants as “a lot” in 39% of cases and as “quite” improvement in 50.6%. The overall level of satisfaction was rated as “very satisfied” by 52.1% of participants and as “quite satisfied” by 40.9%. The results show that the use of dietary supplements to improve different conditions, particularly minor disorders, is a widespread clinical practice among Spanish pediatricians. Administration regimens for the three types of supplements, synbiotics, immune stimulants, and omega-3 PUFAs, were consistent with guideline recommendations.

## 1. Introduction

Dietary supplements in children and adolescents have become very popular and are increasingly used in most industrialized countries. Potential benefits to maintain health and well-being are based on a clear relationship between nutrition and adequate intake of micronutrients, vitamins, minerals, and other trace elements [1, 2], but some features such as accessibility, ease of use, and the belief they are safe without side effects have led to a widespread use of dietary supplement products. Definitions of food supplements, dietary guidelines, and nutrient reference values have been established by most health authorities for policy makers and to help consumers to make healthy dietary choices [3–5]. However, surveys of micronutrient consumption among European pediatric populations revealed a substantial proportion of children whose intake of some vitamins and other elements especially vitamin D, vitamin E, iron, iodine, and folate were below the estimated average requirements [6]. It was shown that nutritional deficiencies concurrent with excess body weight can be present, and that ready access to food does not ensure healthy food choices required for adequate amounts of essential nutrients [6].

There is increasing evidence that some dietary supplements are beneficial for overall health and for managing some health conditions, such as omega-3 polyunsaturated fatty acids (PUFAs), docosahexaenoic acid (DHA), and eicosapentaenoic acid (EPA) on neurodevelopment of healthy children [7–10] and as a treatment option in attention-deficit hyperactivity disorder (ADHD) [11, 12], or the role of probiotic species to modulate the gut microbiota and interact with the immune system [13]. Probiotics can exert pleiotropic effects in the prevention and management of different conditions, including gastroenteritis [14, 15], antibiotic-associated diarrhea [16], allergic disorders [17], atopic dermatitis [18], or respiratory infections [19].

Unfortunately, the increase in the use of dietary supplements in pediatric populations has not been associated with understanding of the properties of supplements and their ingredients, the risks of adverse events, and the potential interactions with drugs [20]. There is a need to inform the general population about the use of dietary supplements and also for doctors to update and disseminate adequate knowledge of supplementation to their patients. Doctors together with friends/relatives and media are one of the most common sources of information regarding dietary supplements [21]. Although recommendations suggest that physicians engage patients about dietary supplements by inquiring about supplement use, evaluating supplements, discussing available safety and efficacy data, and monitoring for adverse events and therapeutic responses [22], these suggestions do not account for potential inadequate physician knowledge about supplements. Also, different studies have shown that community pharmacists' level of the nutritional and dietary supplement knowledge and understanding of their therapeutic effects is generally poor [23, 24], which is also consistent with inadequate physicians' knowledge of dietary supplement regulation and adverse event reporting [25, 26].

On the other hand, there is little data on opinions and practices of pediatricians concerning recommendations and reasons for prescribing dietary supplements in children. Studies focused on vitamin D supplementation have shown that the knowledge of pediatricians and pediatric residents about the use of vitamin D supplement, time for prescribing, and duration of vitamin D supplement needs to be enhanced [27, 28]. This study was designed to collect information on dietary supplementation practices among Spanish pediatricians in routine clinical conditions.

## 2. Methods

### 2.1. Design and Participants

A cross-sectional survey study (the COMPABI Study, acronym for *Complementos Alimenticios y Bienestar Infantil* (Dietary Supplements and Child Well-Being)) was designed to gather information on the use of dietary supplements by specialists in Pediatrics working in the outpatient setting throughout Spain. The objective of the study was to determine the characteristics of use of dietary supplements by Spanish pediatricians, including the decision, indications, and factors considered when recommending dietary supplements, as well as the patient's age at the time of using dietary supplements, the type of supplement recommended, and the duration of treatment.

Candidates to participate in the study were specialists in Pediatrics involved in the care of children attended in public or private consultations throughout Spain. Pediatricians were eligible, provided that they usually take care of a minimum of 10 outpatients on a daily basis. The participants were recruited through invitations to specialists in Pediatrics registered in the database of Laboratorios Ordesa, a pharmaceutical company specialized in infant nutrition and pediatric food supplements. The sample was nonrandomized and proportionally stratified to the number of specialists in Pediatrics registered in the autonomous communities of the country. Participation in the study was anonymous, and voluntary. Pediatricians who met the inclusion criteria and accepted to participate in the study were provided with the data collection logbook.

### 2.2. Study Questionnaire and Data Collection

For the purpose of the study, a team of professionals in the health care and sociology fields with experience in survey studies developed the study questionnaire. The questionnaire consists of two parts. The first part included sociodemographic data of participants and general considerations regarding the use of dietary supplements in clinical practice. For the second part of the questionnaire, participants were requested to describe indications, instructions, and recommendations for the use of three categories of dietary supplements in 10 of their pediatric patients. These included synbiotics (combination of probiotics: *Lactobacillus rhamnosus*, *Lactobacillus helveticus*, *Bifidobacterium infantis* IM1 and prebiotics: fructooligosaccharides); omega-3/6 fatty acids (DHA, EPA, and gamma-linoleic acid (GLA)); and immune stimulants (vitamin C and beta-glucan Imunoglukan).

Data collected included in the first part of the questionnaire were as follows: participants age and gender; type of dietary supplement (categorized as vitamins and/or minerals, omega-3 fatty acids (DHA/EPA), probiotics/prebiotics/synbiotics, immune stimulants, phytotherapy, and others); indication of supplementation (categorized as first choice whenever possible, in case of mild disease, when pharmacological treatment is not effective, combination of pharmacological treatment and dietary supplementation); conditions in which dietary supplements were recommended (i.e., gastrointestinal disorders, combination with antibiotics, stimulation of defenses/cold prevention, improvement of atopy symptoms, concentration, attention-deficit hyperactivity disorder (ADHD) symptoms, nutritional status, cough and mucus, constipation, and others); use of phytotherapy and homeopathy; and factors considered when making a recommendation for using dietary supplements (i.e., confidence and previous experience with the product, composition, and indications of the product, tolerability, ease of taking, parents' preferences/suggestions, price, availability of product samples, and others). For the second part of the questionnaire, the following data were recorded: age and gender of patients; patient's age according to the recommended product; indications and duration of treatment; clinical improvement (categorized as nor at all, slight, quite, and a lot); and physician's satisfaction with the supplement (categorized as slightly satisfied, moderately satisfied, quite satisfied, and very satisfied). For the purpose of the analysis, data of patients treated only with one of the three categories of dietary supplements were assessed.

### 2.3. Statistical Analysis

Categorical variables are expressed as frequencies and percentages and quantitative variables as mean and standard deviation (SD) or median and interquartile range (IQR) (25^th^–75^th^ quartile). Differences in the distribution of percentages of categorical variables were analyzed with the chi-square (*χ*^2^) test or Fisher's exact test according to the conditions of application. Between-group differences of quantitative variables were assessed with one-way analysis of variance (ANOVA), with homogeneity assumption evaluated with Leven's tests and the Brown–Forsythe modification. Post hoc analyses for multiple comparisons were performed using Bonferroni's test or Tamhane's T2 test when equal variables were assumed or not assumed, respectively.

Statistical analyses were performed using IBM SPSS Statistics version 23.0 (IBM, Armonk, NY, USA). Statistical significance was set at *P* < 0.05.

## 3. Results

A total of 433 specialists in Pediatrics (62% men) completed the study questionnaire. The mean age was 52.5 (9.3) years (78.4% of participants were older than 45 years). Results of the first part of the questionnaire are shown in Table 1. Probiotics/prebiotics/synbiotics were used by almost all pediatricians (91.9%) followed by immune stimulants (80.4%), vitamins and/or minerals (76.2%), and omega-3 fatty acids (75.1%). Differences in the use of dietary supplements according to the pediatrician's age were not found, except for immune stimulants that were used by a larger percentage of those >45 years as compared ≤45 years (86.2% vs 69.0%, *P*=0.001). Also, 81.1% of participants decided to use dietary stimulants in combination with pharmacological treatment. In relation to indications of supplementation, vitamins and/or minerals were mainly recommended to improve nutritional status (74.8%), omega-3 fatty acids to improve symptoms of attention-deficit hyperactivity disorder (ADHD) (84.8%) and concentration (80.1%), probiotics/prebiotics/synbiotics in combination with antibiotics (92.6%) and in the presence of gastrointestinal disorders (91.2%), immune stimulants to enhance defenses and cold prevention (87.1%), and phytotherapy to relief cough and reduce mucus secretion (29.3%). Half of participants (54.1%) reported the use of homeopathy in about 10% of their patients.

**Figure 1:**
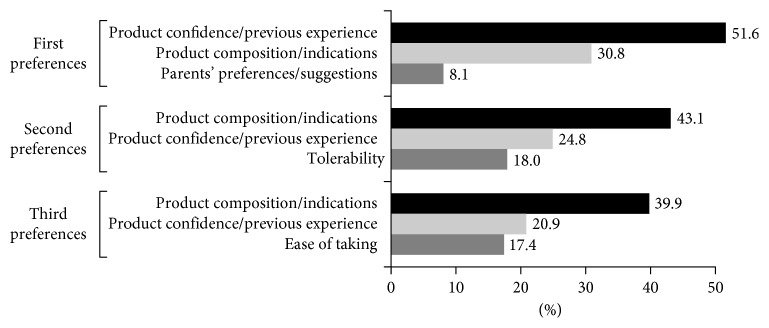
Factors taken into account at the time of recommending a dietary supplement by order of preferences.

The most frequent factors involved in the decision of recommending a dietary supplement were confidence and previous experience with the product (51.6%), composition and indications of the product (43.1%), and tolerability (39.9%) (Figure 1). In the second part of the questionnaire, data of 4.303 pediatric patients treated with dietary supplements of synbiotics, omega-3, and immune stimulants were recorded. The main (SD) age of children was 4.5 (3.6) years. A total of 4074 children (94.7%) were treated only with one of the three categories of dietary supplements. Of these, 1763 (43.3%) received synbiotics, 1457 (35.8%) immune stimulants, and 854 (21.0%) omega-3 fatty acids. As shown in Table 2, there were statistically significant differences among the three dietary supplements in the patient's age, reason for recommendation, and duration of treatment. Children treated with omega-3 supplements were significantly older (mean age 7.6 (3.0) years) than those treated with synbiotics (3.9 (3.9) years) or immune stimulants (3.4 (2.8) years) (*P* < 0.001). Reasons for recommending dietary supplementation also varied significantly among the three groups according to indications of each dietary supplementation category. Short duration of treatment (<1 month) was significantly more common in the synbiotics group (90.5%), whereas longer duration of treatment (>3 months) was more frequent in the omega-3 group (79.1%). In the immune stimulants group, 60.4% of patients were treated for a period between 1 and 3 months.

**Figure 2:**
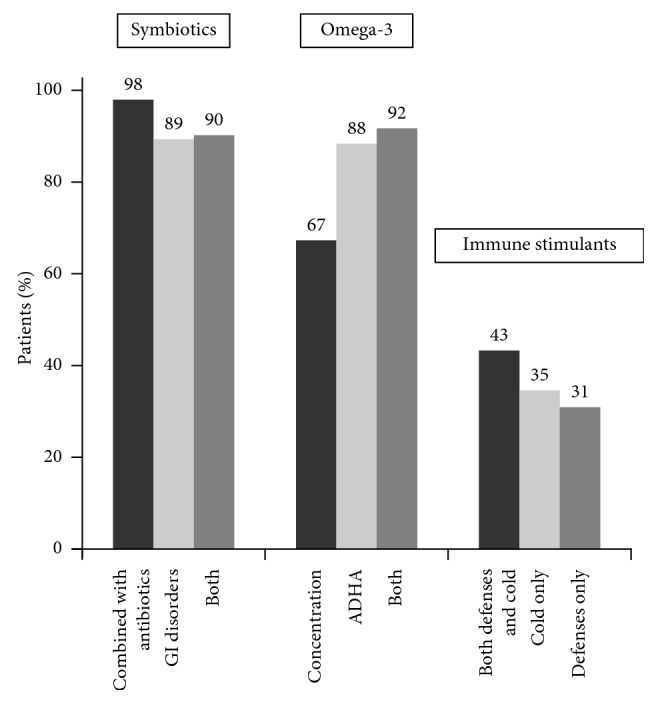
Differences in the percentage of patients treated with synbiotics during 1 month, with omega-3 during 3 months, and with immune stimulants during 3 months according to indications of treatment (GI: gastrointestinal; ADHD: attention-deficit hyperactivity disorder).

Also, the duration of treatment in each category of dietary supplement varied significantly according to main reasons of recommendation (Figure 2). In the group of synbiotics, the percentage of patients treated with synbiotics in combination with antibiotics for 1 month was 98.1% as compared to those in which the reason for prescription was gastrointestinal disorders only (89.2%) or both reasons simultaneously (90.1%) (*P* < 0.001). In the group of omega-3, patients treated for 3 months to improve concentration (67.3%) accounted for a lower percentage versus those treated for ADHD symptoms (88%) or both reasons simultaneously (91.7%) P<0.001. In the group of immune stimulants, the percentage of patients treated for 3 months because of improvement of defenses and/or cold symptoms was higher (43.3%) than those treated for cold symptoms only (34.9%) or to improve defenses only (31.0%) (*P* < 0.001).

Clinical improvement was rated by participants as “a lot” in 39% of cases, “quite” improvement in 50.6%, and “not at all” or “slight” in only 10.4%. There were significant differences according to the category of dietary supplements, with “a lot” of improvement being more frequent in the omega-3 group (Table 2). The overall level of satisfaction was rated as “very satisfied” by 52.1% of participants, “quite satisfied” by 40.9%, and “moderately” or “slightly satisfied” by only 7.1%. Also, the percentages of “very satisfied” was higher for the category of synbiotics (62.7%) as compared to 53% for immune stimulants and 30.2% for omega-3 supplements (*P* < 0.001) (Table 2).

## 4. Discussion

The present study examined the use of dietary supplements by Spanish pediatricians in daily practice. A remarkable finding is the large percentage of users (more than 75%), particularly probiotics/prebiotics/synbiotics, immune stimulants, and omega-3 fatty acids, which is consistent with increase in the use of dietary supplements in the last decades reported in the literature [29, 30]. Interestingly, most participants used dietary supplements in association with pharmacological treatment, and recommendations were based on evidence of their beneficial preventive and therapeutic effects, in contrast to reasons, such as “supplementing the diet” or to “maintain health” when use of supplements is not based on the recommendations of a physician or other health care provider [29]. Of note, the high percentage of pediatricians (54.1%) report the use of homeopathy, which was much greater than those recommending herbal medicinal products (20.3%), despite insufficient evidence and lack of acceptable effectiveness of homeopathy in pediatric patients [31].

Dietary supplements mostly used by pediatricians were synbiotics for the relief of symptoms related to gastrointestinal infections [32] or secondary to the use of antibiotics [33] and to a lesser extent for the enhancing effect on innate immunity [34]. Immune stimulants were used because of the immunomodulatory effect of biologically active polysaccharides. Beta-glucans are one of the most studied natural immunomodulators with confirmed pluripotent biological activities applicable in different clinical situations, both for therapy and prevention, such as respiratory tract infections [35–38] and allergic diseases [39, 40]. With regard to omega-3 fatty acids supplements, the classical indications for this type of products were followed based on the key role of omega-3 PUFAs in brain development and function [41, 42]. A recent systematic review and meta-analysis of seven randomized controlled trials with a total of 534 youth with ADHD and PUFAs supplementation monotherapy improved clinical symptoms and cognitive performance [11].

The recommended duration of treatment with dietary supplements was quite homogenous and agreed with routine regimens of less than 1 month for synbiotics, between 1 and 3 months for immune stimulants and more than 3 months for omega-3 PUFAs. Ages of the patients treated with dietary supplements were also consistent with the occurrence of gastrointestinal disorders in infants and early age, upper respiratory infections at starting school or day care, and ADHD and attention deficits at the age of primary education. The participants' perception of both improvement of clinical symptoms and level of satisfaction was greater for synbiotics as compared to immune stimulants and omega-3 DHA/EPA, which is probably related to the more complex and multifactorial mechanisms underlying ADHD and susceptibility to respiratory infections. Finally, pediatricians reported the confidence and previous experience with the product as the first factor influencing the choice when recommending a dietary supplement.

In the wide context of products known variously as dietary supplements, natural health products, complementary medicines, or food supplements, it is important to distinguish between “traditionally used” and “well-established used” dietary supplements. Some categories of food supplements are only based on what is known as “traditionally used” (in particular, herbal medicinal plants) following the common perception that they are generally beneficial for health and may help child's development and growth but without confirmed efficacy resulting from clinical trials or intervention studies. On the other hand, we can increasingly find more food supplements that can be classified as “well-established used” because their efficacy and safety have been clearly established by appropriate clinical studies and their indications are based in evidence-based medicine. For this reason, in front of the wide offer of pediatric supplements available in the market, especially in some categories, the evaluation of them must be based on their scientific basis, taking into account different clinical studies that demonstrate the efficacy of the main components, dosages, indications, and duration of the treatment. The three food supplements selected for this study (symbiotic, omega-3, and immune stimulants) accomplish with these guidelines and their utilization in pediatrics is well supported by well-designed and valid studies published in the literature [3–5, 8–11, 13–15, 34].

During the last years, in the Spanish pharmaceutical market as well as in other European markets, there has been a wide proliferation of homeopathic preparations with pediatric indications, supported by their safety and excellent tolerance, and in most cases focused to alleviate minor complaints, such as infant colic, discomfort associated with teething or natural defenses stimulation, mostly used since the first months of life. Despite knowing this fact, the high percentage of pediatricians who have mentioned prescribing this type of product has been higher than initially expected. Taking these data into consideration, recent positioning of different scientific societies and national and international health authorities against indiscriminate use of homeopathy should lead to a change in clinical practice due to the scarce scientific basis for the use of this type of products in both young children and adults, in favor to other preparations with greater scientific endorsement such as those with formulas based on phytotherapeutic compounds of recognized composition and efficacy.

The present results should be interpreted taking into account some limitations of the study, especially the fact that a probability or a stratified random sample was not calculated according to the global universe of specialists in pediatrics potentially prescribing dietary supplements to their patients. Therefore, it is not possible to establish the degree of representativeness of the participants in the study. However, all questionnaires were completed in full and no case was excluded because of incomplete data. Adherence to the recommended therapy cannot be evaluated as this topic was not included in the study questionnaire, but the results of clinical improvement and satisfaction indirectly suggest adequate compliance with the recommended therapy. The questionnaire was written in Spanish and distributed nationwide, which eliminates the confounding factor of language barriers and differences related to organizations of different health care systems. The aim of the survey was to provide information on the opinion of pediatricians regarding the use of three common dietary supplements (synbiotics, immune stimulants, and omega-3 DHA/EPA) given to children in daily practice. Despite these limitations, no previous studies have evaluated the use of dietary supplements among Spanish pediatricians, so that our findings present useful information of an extended practice as a starting point for further research.

## 5. Conclusion

This survey shows that the use of dietary supplements to improve different conditions, particularly minor disorders, is a widespread clinical practice among Spanish pediatricians. Administration regimens for the three type of supplements, synbiotics, immune stimulants, and omega-3 PUFAs, followed clinical guideline recommendations. There was a high level of satisfaction regarding improvement of symptoms associated with the use of dietary supplements.

## Figures and Tables

**Table 1:** Type of dietary supplement, indications, and factors considered at the time of prescription in the survey of 433 pediatricians.

Item of the questionnaire	Number (%)
What type of pediatric dietary supplements do you usually use?	
Vitamins and/or minerals	330 (76.2)
Omega-3 fatty acids (DHA/EPA)	325 (75.1)
Probiotics/prebiotics/synbiotics	398 (91.9)
Immune stimulants	348 (80.4)
Phytotherapy in general	88 (20.3)
Others	19 (4.4)
When do you decide to use a dietary supplement?	
As a first choice whenever possible	134 (30.9)
In case of a mild disease	172 (39.7)
When pharmacological treatment is not effective?	64 (14.8)
I use to combine pharmacological treatment and dietary supplementation	351 (81.1)
On parent's request	93 (21.5)
In which of conditions do you recommend the following dietary supplements?	
Vitamins and/or minerals	
Improvement of nutritional status	324 (74.8)
Stimulation of defenses/cold prevention	71 (16.4)
Improvement of concentration	55 (12.7)
Improvement of ADHD symptoms	30 (6.9)
Omega-3 fatty acids	
Improvement of ADHD symptoms	367 (84.8)
Improvement of concentration	347 (80.1)
Improvement of atopy symptoms	81 (18.7)
Improvement of nutritional status	64 (14.8)
Probiotics/prebiotics/synbiotics	
Combination with antibiotics	401 (92.6)
Gastrointestinal disorders	395 (91.2)
Constipation	221 (51.0)
Improvement of atopy symptoms	123 (28.4)
Immune stimulants	
Stimulation of defenses/cold prevention	377 (87.1)
Cough and mucus	182 (42.0)
Improvement of atopy symptoms	89 (20.6)
Improvement of nutritional status	43 (9.9)
Phytotherapy	
Cough and mucus	127 (29.3)
Constipation	81 (18.7)
Gastrointestinal disorders	39 (9.0)
Improvement of atopy symptoms	39 (9.0)
Do you use homeopathy?	
Yes	226 (54.1)
No	192 (45.9)
Missing	15 (3.5)

DHA: docosahexaenoic acid; EPA: eicosapentaenoic acid; ADHS: attention-deficit hyperactivity disorder.

**Table 2:** Characteristics of the use of three categories of dietary supplements.

Variables	Synbiotics (*n* = 1763)	Omega-3 (*n* = 854)	Immune stimulants (*n* = 1457)	*P* value
Children's age, years, mean (SD)	3.9 (3.9)	7.6 (3.0)	3.4 (2.8)	<0.001
Reasons for the recommendation				
Gastrointestinal disorders	1223 (99.4)	0	8 (0.6)	<0.001
Combination with antibiotics	759 (93.7)	0	51 (6.3)	<0.001
Stimulation of defenses	22 (1.8)	2 (0.2)	1232 (98.1)	<0.001
Cold prevention	11 (1.2)	4 (0.4)	889 (98.3)	<0.001
Improvement of atopy symptoms	29 (21.2)	58 (42.3)	50 (36.5)	<0.001
Improvement of concentration	2 (0.3)	585 (99.0)	4 (0.7)	<0.001
Improvement of ADHD symptoms	4 (0.8)	467 (98.9)	1 (0.2)	<0.001
Duration of treatment				
<1 month	1580 (90.5)	7 (0.8)	30 (2.1)	<0.001
1–3 months	126 (7.2)	170 (20.0)	875 (60.4)	<0.001
>3 months	39 (2.2)	671 (79.1)	544 (37.5)	<0.001
Clinical improvement (%)				
Not at all	0.4	2.2	0.6	NS
Slight	3.4	24.8	7.1	*P* < 0.001
Quite	40.5	54.7	58.5	*P* < 0.001
A lot	55.7	18.3	33.8	*P* < 0.001
Level of satisfaction (%)				
Slightly satisfied	0.4	2.2	0.4	<0.001
Moderately satisfied	2.3	19.4	3.9	<0.001
Quite satisfied	34.7	48.2	42.6	<0.001
Very satisfied	62.7	30.2	53.0	<0.001

## Data Availability

The data used to support the findings of this study are available from the corresponding author upon request.
